# Additive genetic variance and covariance between relatives in synthetic wheat crosses with variable parental ploidy levels

**DOI:** 10.1093/genetics/iyaa048

**Published:** 2021-01-07

**Authors:** L E Puhl, J Crossa, S Munilla, P Pérez-Rodríguez, R J C Cantet

**Affiliations:** 1 Departamento de Métodos Cuantitativos y Sistemas de Información, Facultad de Agronomía, Universidad de Buenos Aires, 1417 Ciudad Autónoma de Buenos Aires, Argentina; 2 Biometrics and Statistics Unit. International Maize and Wheat Improvement Center (CIMMYT), Carretera México -Veracruz, Km 45, Col. El Batán, CP 56237, Texcoco, Edo. de México, México; 3 Departamento de Estadística, Colegio de Postgraduados, Montecillo, Edo. de México, CP. 56230, México; 4 Departamento de Producción Animal, Facultad de Agronomía, Universidad de Buenos Aires. Instituto de Investigaciones en Producción Animal (INPA), Consejo Nacional de Investigaciones Científicas y Técnicas, 1417 Ciudad Autónoma de Buenos Aires, Argentina

**Keywords:** synthetic wheat, polyploidy, additive genetic variance, breeding values

## Abstract

Cultivated bread wheat (*Triticum aestivum* L.) is an allohexaploid species resulting from the natural hybridization and chromosome doubling of allotetraploid durum wheat (*T. turgidum*) and a diploid goatgrass *Aegilops tauschii* Coss (*Ae. tauschii*). Synthetic hexaploid wheat (*SHW*) was developed through the interspecific hybridization of *Ae. tauschii* and *T. turgidum*, and then crossed to *T. aestivum* to produce synthetic hexaploid wheat derivatives (*SHWDs*). Owing to this founding variability, one may infer that the genetic variances of native wild populations *vs* improved wheat may vary due to their differential origin and evolutionary history. In this study, we partitioned the additive variance of *SHW* and *SHWD* with respect to their breed origin by fitting a hierarchical Bayesian model with heterogeneous covariance structure for breeding values to estimate variance components for each breed category, and segregation variance. Two data sets were used to test the proposed hierarchical Bayesian model, one from a multi-year multi-location field trial of *SHWD* and the other comprising the two species of *SHW*. For the *SHWD*, the Bayesian estimates of additive variances of grain yield from each breed category were similar for *T. turgidum* and *Ae. tauschii*, but smaller for *T. aestivum*. Segregation variances between *Ae. tauschii—T. aestivum* and *T. turgidum—T. aestivum* populations explained a sizable proportion of the phenotypic variance. Bayesian additive variance components and the Best Linear Unbiased Predictors (BLUPs) estimated by two well-known software programs were similar for multi-breed origin and for the sum of the breeding values by origin for both data sets. Our results support the suitability of models with heterogeneous additive genetic variances to predict breeding values in wheat crosses with variable ploidy levels.

## Introduction 

Wheat is a popular staple crop that adapts well to different vernalization and photoperiod environments as well as to salt, winter frost, and aluminum conditions. Cultivated wheat (*Triticum aestivum*) is an allohexaploid (2*n* = 42) with three genomes, A, B, and D, each with seven chromosomes and with a large genome of 16 × 10^9^ bp (Bennett and Smith 1976). Bread wheat results from the natural hybridization and chromosome doubling of two populations with different ploidy: a cultivated allotetraploid durum wheat (*T. turgidum;* 2*n* = 4x = 28 tetraploid genome AABB) and a diploid goatgrass *Aegilops tauschii* Coss. (*Ae. tauschii* Coss.; 2n = 2x = 14 diploid genome DD). When these crossings occurred is not well known, but they most probably involved spontaneous and casual crossings of a few individuals from two distantly related grasses. Simonite (2006) suggested that only one such hybridization is still represented in modern wheat and, because these crossings involved few progenitors, the genetic diversity of durum wheat and *Ae. tauschii* is not well represented in wheat germplasm (Dreisigacker *et al.* 2008; Li *et al.* 2014).

Due to the intense selection pressure to increase crop yield and stability, genetic variation in bread wheat (*T. aestivum*, hexaploid genome AABBDD) has been reduced (Reif *et al.* 2005; Warburton *et al.* 2006; Jafarzadeh *et al.* 2016). The peculiar origin of bread wheat allowed the development of synthetic hexaploid wheat lines (*SHW*) (Cox *et al.* 1995; Mujeeb-Kazi *et al.* 1996), which resulted in an artificial recreation of the original crossing process between the diploid and tetraploid parents. The goal was to introduce new genetic diversity into the already improved and highly productive bread wheat, by crossing and backcrossing synthetic lines with *T. aestivum*, which resulted in synthetic hexaploid wheat derivative lines (*SHWDs*). This artificially created hexaploid represents a wider genetic diversity gene pool that should enable wheat researchers to introduce novel genetic variation lost during the process of origin and domestication of wheat (Li *et al.* 2018).

As mentioned above, bread wheat is the result of crossing diverse wheat species with different ploidy and evolutionary history, whose progeny will carry portions of the genomes of both species, *T. turgidum* and *Ae. tauschii*, according to the ploidy and composition of their ancestors. Indeed, it is natural to think that for any trait of interest, the genetic variance of native wild populations and improved wheat may be different. Molecular studies have revealed a significant increase in the genetic diversity of wheat lines, including synthetic wheat derivative lines, compared to traditional wheat cultivars (Warburton *et al.* 2006; Dreisigacker *et al.* 2008). Most wheat diversity comes from its diploid and tetraploid parents, as observed in the outcome of several specific traits (Cox *et al.* 1995) and as registered at the DNA sequence level (Lage *et al.* 2003; Zhang *et al.* 2005; Jafarzadeh *et al.* 2016). In traits related to biotic and abiotic stress, Jighly *et al.* (2018) discovered that the D subgenome contributed more to the total additive variance than the A and B subgenomes in a synthetic wheat population.

Wheat synthetic hybrids have many unfavorable traits in comparison with ordinary wheat lines. Their plants are normally taller, difficult to thresh, late maturing, and have reduced biomass and yield; this is why desirable traits that could be transmitted to their progeny are not easy to identify in their own phenotype (Mujeeb-Kazi *et al.* 1996). Nevertheless, synthetic derivative lines have been exhaustively evaluated and have shown resistance to major wheat diseases and tolerance to both biotic stresses (such as pre-harvest sprouting) and abiotic stresses (such as drought, heat, waterlogging, and salinity; van Ginkel and Ogbonnaya 2007), surpassing the performance and resistance of the local varieties used as checks. Determining the breeding value of synthetic wheat lines based on the performance of their progeny allows calculating the genetic merit of the parents indirectly.

Nowadays the Best Linear Unbiased Predictor methodology (BLUP; Henderson 1975) is widely used to predict the genetic merit or breeding value of wheat cultivars (Crossa *et al.* 2006; Piepho *et al.* 2008; Xavier *et al.* 2016). BLUP’s predictions are obtained by fitting a linear mixed model to performance data. Under this approach, the breeding values (***a***) are treated as a random effect with a covariance matrix defined by $\mathrm{Var}\left(a\right)=A\sigma_{A}^{2}$, where ***A*** represents the numerator (additive genetic) relationship matrix (Wright 1922), which is computed based on the known pedigree, and $\sigma_{A\;}^{2}$ denotes the additive genetic variance. This latter parameter, as well as other variance components, are assumed known when setting up and solving Henderson’s mixed model equations (Henderson 1984) to compute the breeding values. The method most widely used for estimating variance components is restricted maximum likelihood (REML, Patterson and Thompson 1971). There is also a rich body of literature dealing with the use of the Gibbs sampling algorithm to estimate these variance parameters in the framework of a Bayesian hierarchical approach (Wang *et al.* 1993; Sorensen and Gianola 2002).

The standard model used to predict breeding values of synthetic wheat lines and their progenitors for variety selection assumes one breeding population and a single additive genetic variance parameter for all wheat populations involved (Crossa *et al.* 2006; Dreccer *et al.* 2007; Jafarzadeh *et al.* 2016). However, considering the available evidence about the irregular genetic diversity across wheat populations, a mixed model for predicting breeding values of synthetic derivatives should consider heterogeneous additive genetic variances according to their breed’s origin: *Ae. tauschii*, *T. turgidum* and *T. aestivum*. It has been observed that assuming equal variance across breeding groups is not appropriate for a multibreed population (Elzo and Bradford 1985; Elzo 1990; Wei *et al.* 1991). Breeding values should be expressed relative to their respective breed of origin, assuming a substructure associated with their variance (which is associated with specific base populations) and concomitant relationship matrices. Lo *et al.* (1993) described how to obtain the covariance matrix of the breeding values including not only the contribution of each individual breed but also the segregation component that explains the differences in the additive variance of the F_1_ and F_2_ segregating populations. Subsequently, García-Cortés and Toro (2006) presented an equivalent model to account for this covariance structure based on splitting the breeding values into independent components by genetic origin. In the latter model, each component has its own covariance matrix, defined by a ‘partial’ numerator relationship matrix times a corresponding variance component. The term ‘partial’ is related to the fact that only the corresponding source of variability is considered when setting-up this matrix (García-Cortés and Toro 2006).

Considering the decomposition of the multi-breed genetic covariance matrix described by García-Cortés and Toro (2006), the goals of this research were to: (1) develop a general approach to compute the covariance structure of breeding values for *SHW* and *SHWD* wheat lines while taking into account variable ploidy levels; (2) estimate the additive genetic variances of the three populations comprising the synthetic wheat, as well as the extra variances that result from crossing them; and (3) predict breeding values of both *SHW* and *SHWD* data sets using two different well-known software programs when considering the multi-breed origin versus considering only one-breed and comparing their results.

Throughout the years, the International Maize and Wheat Improvement Center (CIMMYT) has produced thousands of *SHW* and *SHWD* lines. In this study, two data sets comprising *SHW* and *SHWD* were used. Data set 1 included 13 cycles (1997-2010) of the Semi-Arid Wheat Yield Trial (SAWYT) from the Global Wheat Breeding Program of CIMMYT. The data consisted of *SHW*D wheat yield trials in dryland environments with breeds denoted as diploid *Ae. tauschii* (*D*), tetraploid *T. turgidum* (*T*), and hexaploid *T. aestivum* (*V*) of *SHWD*. The total number of locations reporting data was 170. Data set 2 had wheat synthetic lines (*SHW*) derived from 422 crosses between *Ae. tauschii* (*D*) and *T. turgidum* (*T*) evaluated in one environment for one disease.

For comparing results, we used two well-known general-purpose software programs for fitting hierarchical Bayesian models with pedigree information: the BGLR package (Pérez and de los Campos 2014) and Stan R software (https://mc-stan.org) (Carpenter *et al.* 2017).

## Models and methods

### The hierarchical Bayesian model with pedigree information

#### Genotypic variances for the different breed groups

**Figure 1:**
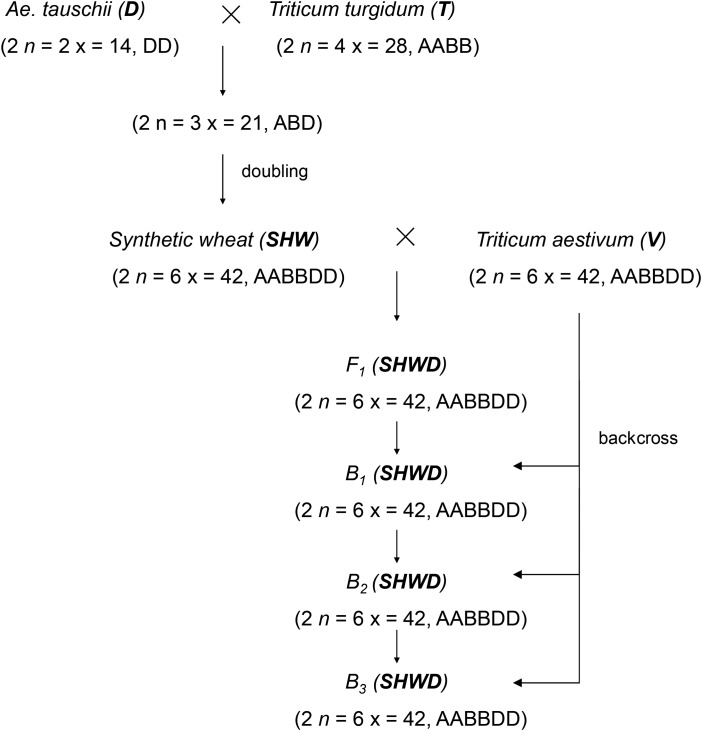
Synthetics (SHW) and synthetic derivative breed crosses (SHWD). Scheme of the breeding system for producing synthetic wheat and its derivatives.

Assume a population with individuals pertaining to one of the several breed groups described in Figure 1. As already mentioned, breed groups are denoted as follows: *D*: diploid *Ae. tauschii*; *T:* tetraploid *T. turgidum*; *V*: hexaploid *T. aestivum*; *SHW*: Synthetic Hexaploid Wheat lines and *SHWD*: Synthetic Hexaploid Wheat Derivative lines. It is important to note that *T*, *V*, *SHW* and *SHWD* breed groups are allopolyploids, a process that involves the merging of fully distinct genomes. Therefore, pairing behavior during meiosis is expected to resemble pairing behavior in diploids, and inheritance can be considered independent among genomes. Let us also assume the trait of interest is under the influence of a large number of unlinked loci that act additively and, thus, the genotypic value of any locus in individual *i* can be modeled by:

(1) $$G_{i}^{BG}=\mu +\sum_{j=1}^{p_{1}}\alpha_{P_{ij}}+\sum_{k=1}^{p_{2}}\alpha_{M_{ik}},$$

where $\alpha_{P_{ij}}$ and $\alpha_{M_{ik}}$ are, respectively, the additive effects of the alleles inherited from the “paternal” (*P_i_*) and “maternal” (*M_i_*) breed group, and *p*_1_ and *p*_2_ stand for the ploidy of these groups. For example, if the paternal line is *Ae. tauschii*, a diploid species, it will only contribute one allele (*p*_1_ = 1) and its effect will be denoted as $\alpha_{P_{i1}}$. In this context, Lo *et al.* (1993) derived the genotypic variance as the sum of the variances of the alleles’ effects in the pure breed groups, each multiplied by the respective probability of the allele coming from this pure breed group, plus a segregation variance that arises due to differences in allelic frequencies between these groups. First, we derive the genotypic variance of the pure breeds, the synthetic hybrids and the synthetic F_1_ derivatives. Next, we use Lo *et al.* (1993) formulae to obtain the genotypic variance of a backcross between a synthetic derivative line to *T. aestivum*.

#### Pure breeds

Starting with diploid *Ae. tauschii* and adapting expression (1) (notice a superscript is added to indicate the breed group of the inherited allele), the genotypic value is:

(2) $$G_{i}^{D}=\mu +\alpha_{P_{i1}}^{D}+\alpha_{M_{i1}}^{D}.$$

Similarly, genotypic values for *T. turgidum* (tetraploid) and *T. aestivum* (hexaploid) are:

(3) $$G_{i}^{T}=\mu +\alpha_{P_{i1}}^{T}+\alpha_{P_{i2}}^{T}+\alpha_{M_{i1}}^{T}+\alpha_{M_{i2}}^{T}$$

 

(4) $$G_{i}^{V}=\mu +\alpha_{P_{i1}}^{V}+\alpha_{P_{i2}}^{V}+\alpha_{P_{i3}}^{V}+\alpha_{M_{i1}}^{V}+\alpha_{M_{i2}}^{V}+\alpha_{M_{i3}}^{V}.$$

In these cases, all individuals are pure breeds and the genotypic variance is a function of a single parameter, the additive genetic variance of the pure breed. In addition, one may consider inbreeding within pure lines. Let $F_{i}^{PB}$ denote the inbreeding coefficient of individual *i* within pure breed *PB* (*PB* = *D*, *T* or *V*), *i.e*., the probability that at any locus the paternal and maternal gametes are identical by descent (IBD, Malecot 1948). Then, when defining the additive variance of allele effects based on ploidy as in Kempthorne (1957) and denoting $\sigma_{A,D}^{2}$, $\sigma_{A,T}^{2}$, and $\sigma_{A,V}^{2}$ as the additive (*A*) variance in *D*, *T* and *V* breed groups, respectively,

(5) $$\sigma_{A,D}^{2}=2\;\mathrm{Var}\left(\alpha_{i}^{D}\right),\;\;\;\sigma_{A,T}^{2}=4\;\mathrm{Var}\left(\alpha_{i}^{T}\right)\;\;\;\text{and}\;\;\;\;\sigma_{A,V}^{2}=6\;\mathrm{Var}\left(\alpha_{i}^{V}\right).$$

Hence, the additive genotypic variances for the pure breeds become:

(6) $$\begin{array}{c} \text{Var}\left(G_{i}^{D}\right)=\text{Var}\left(\mu +\alpha_{P_{i1}}^{D}+\alpha_{M_{i1}}^{D}\right)\\ =\text{Var}\left(\alpha_{P_{i1}}^{D}\right)+\text{Var}\left(\alpha_{M_{i1}}^{D}\right)+2\text{Cov}\left(\alpha_{P_{i1}}^{D},\alpha_{M_{i1}}^{D}\right)\\ =2\text{Var}\left(\alpha_{i}^{D}\right)+2F_{i}^{D}\text{Var}\left(\alpha_{i}^{D}\right)\\ =\sigma_{A,D}^{2}+\sigma_{A,D}^{2}F_{i}^{D}\\ =\left(1+F_{i}^{D}\right)\sigma_{A,D}^{2} \end{array}.$$

Equivalent derivations, assuming only alleles from different gametes may be IBD, lead to:

(7) $$\text{Var}\left(G_{i}^{T}\right)=\left(1+F_{i}^{T}\right)\sigma_{A,T}^{2}$$

 

(8) $$\text{Var}\left(G_{i}^{V}\right)=\left(1+F_{i}^{V}\right)\sigma_{A,V}^{2}.$$

#### Synthetic wheat

Now consider the synthetic derivative wheat produced after doubling chromosomes in an *Ae. tauschii* (*D*) × *T. turgidum* (*T*) hybrid line. Using expression (1), the genotypic value $G_{i}^{S}$ of individual *i* belonging to this *synthetic* (*SHW*) breed group could be represented as:

(9) $$G_{i}^{S}=\mu +\alpha_{P_{i1}}^{D}+\alpha_{P_{i1}}^{D}+\alpha_{M_{i1}}^{T}+\alpha_{M_{i1}}^{T}+\alpha_{M_{i2}}^{T}+\alpha_{M_{i2}}^{T}$$

where $\alpha_{P_{i1}}^{D}$ and $\alpha_{M_{ij}}^{T}$, *j *=* *1 or 2, are, respectively, the additive effects of paternal (*P_i_*) and maternal (*M_i_*) alleles in the breed group *D* or *T* denoted in the superscript. We will assume that diploid *Ae. tauschii (D)* is the male parent and tetraploid *T. turgidum* (*T*) is the female parent. As the synthetic lines were produced by two distinct alleles from *T. turgidum*, there are two different code numbers in the alleles inherited from tetraploid *T. turgidum* maternal alleles. In fact, because the three inherited alleles were doubled (either induced by colchicine or spontaneously), they are identical to their copies and have the same code number.

Applying variance operator rules, the genotypic variance of a synthetic line *i* can be obtained as follows:

(10) $$\begin{array}{c} \text{Var}\left(G_{i}^{S}\right)=\text{Var}\left(\mu +\alpha_{P_{i1}}^{D}+\alpha_{P_{i1}}^{D}+\alpha_{M_{i1}}^{T}+\alpha_{M_{i1}}^{T}+\alpha_{M_{i2}}^{T}+\alpha_{M_{i2}}^{T}\right)\\ =2\text{Var}\left(\alpha_{P_{i1}}^{D}\right)+2\;\text{Cov}\left(\alpha_{P_{i1}}^{D},\alpha_{P_{i1}}^{D}\right)+2\text{Var}\left(\alpha_{M_{i1}}^{T}\right)+\\ +2\;\text{Cov}\left(\alpha_{M_{i1}}^{T},\alpha_{M_{i1}}^{T}\right)+2\text{Var}\left(\alpha_{M_{i2}}^{T}\right)+2\;\text{Cov}\left(\alpha_{M_{i2}}^{T},\alpha_{M_{i2}}^{T}\right)+\\ +8\;\text{Cov}\left(\alpha_{M_{i1}}^{T},\alpha_{M_{i2}}^{T}\right) \end{array}$$

Alleles in different breed groups (*D* and *T*) are independent; therefore, the covariance between their additive effects is null and has been excluded from expression (10) and all further developments. Now, effects $\alpha_{M_{\mathrm{i1}}}^{T}$ and $\alpha_{M_{\mathrm{i2}}}^{T}$ are sampled from the same locus and have the same variance, *i.e*., $\mathrm{Var}\left(\alpha_{M_{\mathrm{i1}}}^{T}\right)=\mathrm{Var}\left(\alpha_{M_{\mathrm{i2}}}^{T}\right)$. In addition, $\alpha_{M_{\mathrm{i1}}}^{T}$ and $\alpha_{M_{\mathrm{i2}}}^{T}$ have very low probability of being IBD because as *T. turgidum* is allopolyploid, these effects originate in different genomes; therefore, they can be assumed independent and the last term in expression (10) is equal to zero. By gathering these results, using definitions in expression (5) and dropping unnecessary subscripts, we can express expression (10) as:

(11) $$\begin{array}{c} \text{Var}\left(G_{i}^{S}\right)=2\text{Var}\left(\alpha_{P_{i1}}^{D}\right)+2\text{Var}\left(\alpha_{P_{i1}}^{D}\right)F_{i}^{D}+\\ +4\text{Var}\left(\alpha_{M_{i1}}^{T}\right)+2\;\text{Var}\left(\alpha_{M_{i1}}^{T}\right)F_{i}^{T}+2\;\text{Var}\left(\alpha_{M_{i2}}^{T}\right)F_{i}^{T}\\ =2\left(1+F_{i}^{D}\right)\text{Var}\left(\alpha_{P_{i}}^{D}\right)+4\left(1+F_{i}^{T}\right)\text{Var}\left(\alpha_{M_{i}}^{T}\right)\\ =\left(1+F_{i}^{D}\right)\sigma_{A,D}^{2}+\left(1+F_{i}^{T}\right)\sigma_{A,T}^{2} \end{array}.$$

Note that the additive variance of synthetic hexaploid lines is the sum of the additive variance of the diploid and tetraploid pure breed groups. Complete derivations of the expressions for the additive variance of the derivatives breed groups are displayed in Appendix 1. Table 1 summarizes the rules to compute the additive variance for each breed group. For synthetic derivatives lines, we define the $f_{i}^{\mathrm{BG}}$ coefficient of an offspring *i* as the average of the breed composition in the paternal and maternal breed groups, *e.g*., $f_{i}^{V}=\frac{1}{2}\left(f_{P_{i}}^{V}+f_{M_{i}}^{V}\right)$ for the *T. aestivum* breed group.

**Table 1:** Additive variances of breed groups for synthetic wheat and derivatives

Breed group	Variance	$f_{i}^{BG}$
Pure breed		
* Ae. tauschii* (diploid) (*D*)	$\left(1+F_{i}^{D}\right)\sigma_{A,D}^{2}$	$f_{i}^{D}=1$
* T. turgidum* (tetraploid) (*T*)	$\left(1+F_{i}^{T}\right)\sigma_{A,T}^{2}$	$f_{i}^{T}=1$
* T. aestivum* (hexaploid) (*V*)	$\;\left(1+F_{i}^{V}\right)\sigma_{A,V}^{2}$	$f_{i}^{V}=1$
Synthetic wheat (hexaploid) (*SHW*)	$\left(1+F_{i}^{D}\right)\sigma_{A,D}^{2}+\left(1+F_{i}^{T}\right)\sigma_{A,T}^{2}$	$f_{i}^{D}=f_{i}^{T}=1$
Synthetic wheat derivatives (hexaploid) (*SHWD*)	$\begin{array}{c} f_{i}^{D}\sigma_{A,D}^{2}+f_{i}^{T}\sigma_{A,T}^{2}+f_{i}^{V}\sigma_{A,V}^{2}+2f_{P_{i}}^{D}f_{P_{i}}^{V}\sigma_{S_{DV}}^{2}\\ +4f_{P_{i}}^{T}f_{P_{i}}^{V}\sigma_{S_{TV}}^{2}+\frac{1}{2}\;\mathrm{Cov}\left(G_{P_{i}},\;G_{M_{i}}\right) \end{array}$	$f_{i}^{\mathrm{BG}}=\frac{1}{2}\left(f_{P_{i}}^{\mathrm{BG}}+f_{M_{i}}^{\mathrm{BG}}\right)$

Synthetic derivatives (*SHWD*) include the cross **F_1_** (***S ***×*** V***) and successive backcrossing to *T. aestivum*. Breed groups are denoted by *BG* with *BG* = (*D*, *T*, *V*, *S*, *SD*). $f_{i}^{\mathrm{BG}}$are coefficients that express the expected proportion of *D*, *T* and *V* genes in individual *i.*

### Covariance between wheat synthetic crossbreed relatives

As shown by Lo *et al.* (1993), covariance between crossbreed relatives can be computed using formulae for purebred populations, if the variance of a crossbreed individual is computed as presented here. We express the variance of a crossbreed individual by splitting the total variance into pure breed variabilities (*D*, *T*, and *V*) and segregation terms (*DV* and *TV*) (see Appendix 1). The $f_{i}^{\mathrm{BG}}$ coefficients described above express the proportion of pure breed variance and segregation terms that make up each crossbreed and determine the diagonal elements of ***A****_Q_*. ***A****_Q_* are the so-called partial numerator additive relationship matrices (García-Cortés and Toro 2006) associated with the dispersion parameters $\sigma_{A,Q}^{2}$ from breed Q with Q = {*D*, *T*, *V*, *DV*, *TV*}. *Q* includes pure breed variabilities *D*, *T* and *V* and segregation terms *DV* and *DT.* The off-diagonal elements of ***A****_Q_* are the relationship coefficients related to covariances between breeding values and are obtained by a tabular method following the rules summarized in Table 1 to obtain the diagonal elements and expressions (12). Overall, the covariance between breeding values for genotypes *i* and *i*’ is equal to:

(12) $$\mathrm{Cov}\left(G_{i},G_{i^{\prime }}\right)=\frac{1}{2}\left[\mathrm{Cov}\left(G_{j},G_{i^{\prime }}\right)+\mathrm{Cov}\left(G_{k},G_{i^{\prime }}\right)\right],$$

where *j* and *k* are the parents of *i*. Expression (12) was obtained by Lo *et al.* (1993) [their expression (10)].

#### Hierarchical Bayesian model and analysis for a multi breed synthetic wheat model

To fit the additive genetic covariance structure just described, we defined a linear mixed model with several random genetic effects, *i.e*., the breeding values by breed origin, ***a***_*Q*_, with *Q = {D, T, V, DV, TV}*, where *D, T*, and *V* are *Ae. tauschii (D)*, *T. turgidum (T)* and *T. aestivum (V)* breed groups, and *DV* and *TV* indicate the segregation effects between *Ae. tauschii- T. aestivum* and *T. turgidum—T. aestivum* populations. Segregation effects were added to the model to account for the segregation variance, defined as the additional genetic variance in an F_2_ population over that in the F_1_ population (Wright 1968; Lande 1981). The model equation is as follows:

(13) $$y=\mathrm{Xb}+\sum_{Q}Z_{Q}a_{Q}+Z_{GE}\mathrm{ge}+e,$$

where ***y*** is the phenotypic data vector and **X** represents the full rank incidence matrix of fixed effects in vector **b**. In this model, **b** represents the fixed effects of the environment corresponding to all location × cycle combinations. Matrices ***Z****_D_*,***Z***_*T*_ and ***Z****_V_* are the incidence matrices for the breeding values (***a***_*D*_, ***a***_*T*_ and***a***_*V*_) of the *Ae. tauschii*, *T. turgidum* and *T. aestivum* breed groups, respectively. Matrices ***Z***_*DV*_ and ***Z****_TV_* are, respectively, the incidence matrices for the segregation random effects (***a***_*DV*_ and ***a***_*TV*_) between *Ae. tauschii* and *T. aestivum*, and between *T. turgidum* and *T. aestivum*. The random effect of the interaction between genotype and environment is modeled in vector ***ge***, whereas ***Z***_*GE*_ is the corresponding incidence matrix. Finally, ***e*** (*n *×* *1) is the error vector. The main objective was to make inferences about the parameters of the model, especially the additive and segregation genetic variances.

The Bayesian inference approach employed to fit model (13) can be described as a hierarchical construction (Munilla-Leguizamón and Cantet 2010; Sorensen and Gianola 2002) and is similar to that employed by García-Cortés and Toro (2006) considering the variance of the individual groups, as well as the difference in the additive variance of the F_1_ and F_2_ groups. Initially, it is necessary to specify the full conditional sampling density of the phenotypic data vector. Following Cantet *et al.* (1992) and assuming a multivariate Normal distribution for ***y***, we have:

(14) $$y|\;b,\;a_{Q},\;\mathrm{ge},\;\sigma_{e}^{2}∼N\left(\mathrm{Xb}+\sum_{Q}Z_{Q}a_{Q}+Z_{GE}\mathrm{ge},\;I_{n}\sigma_{e}^{2}\right).$$

We will now describe the prior distribution assigned to each of the location parameters, **b**, ***a***_Q_ and ***ge***.

We assigned a multivariate Normal distribution to the vector of fixed effects **b** with very large variance to avoid the occurrence of an improper posterior distribution, a problem that appears when a Uniform prior is employed for **b** (Hobert and Casella 1996). That is:

(15) b ‖ K∼N(0, K),

where ***K*** = Diag {***k***_i_}, with ***k****_i_* > 10^7^ with *i *=* *1,…,*p*, where *p* is the number of fixed effects.

In turn, and based on the quantitative genetics theory (Bulmer 1985, Chapter 8), we specified a multivariate Normal distribution for the vector of non-zero breeding values ***a***_*Q*_ corresponding to breed origin *Q*. Symbolically and explicitly, density functions are as follows:

(16) $$a_{Q}\;\|\;\;A_{Q},\;\sigma_{\mathrm{A,Q}}^{2}∼N\left(0\;,\;A_{Q}\sigma_{\mathrm{A,Q}}^{2}\right)$$

 

(17) $$p\left(a_{Q}\;\|\;A_{Q}\right)\;\propto \;\;\exp\left\{-\frac{1}{2\;\sigma_{\mathrm{A,Q}}^{2}}\;\;a_{Q}^{\prime }A_{Q}^{-1}a_{Q}\right\},$$

where ***A***_*Q*_ are the so-called partial numerator additive relationship matrices (García-Cortés and Toro 2006) associated with the dispersion parameters $\sigma_{A,Q}^{2}$ from breed *Q*. The entries of these matrices are the relationship coefficients between breeding values according to breed origin and the methodology to compute them was described above.

Finally, the random vectors of genotype by environment interaction ***ge*** effects were assumed to be independent and follow a multivariate Normal distribution, such that:

(18) $$\mathrm{ge}\;\|\;\sigma_{\mathrm{GE}}^{2}∼N\left(0,\;I\sigma_{\mathrm{GE}}^{2}\right).$$

In the next level of the hierarchy, dispersion parameters are assigned inverse scaled chi-squared distributions (see details in Appendix 2). Subsequently, by combining the likelihood and the conjugate prior distributions, one can obtain the joint posterior distribution of all the unknowns of the model. Finally, marginalization of this latter distribution to obtain samples of the parameters of interest is performed by means of a standard Gibbs sampler (*e.g*., Sorensen and Gianola 2002). This is feasible, because all conditional posterior distributions have closed form. The explicit form of the joint posterior distribution and the full conditional distribution of the parameters of interest are presented in detail in Appendix 2.

## Experimental data

### *Data set 1* (includes synthetic derivatives wheat lines, SHWD)

Data on 13 cycles (1997–2010) of the SAWYT (Semi-Arid Wheat Yield Trial) were available for this study. The data consisted of wheat yield trials in dryland environments that belong to international CIMMYT collaborators. The experiments were arranged in an incomplete randomized block design with two replicates. Synthetic derivative lines were progressively included in SAWYT trials as they developed, reaching 46% of the wheat lines tested in 2006. However, most of them were not repeated in consecutive years, causing high temporal discontinuity in their evaluations (Figure 1, lower diagonal).

Overall, 97% of the genotypes were evaluated in a single cycle. For this study, we chose all the synthetic derivatives from the SAWYT, resulting in 88 genotypes, all with phenotypic data. Almost all of these genotypes were present in every location in a given year. The main imbalance was generated in successive years, in which both genotypes and locations were not repeated, or only partially repeated. The total number of locations reporting data was 170, 61% of which were not repeated in different years, meaning that they were incorporated as sites only once. The number of common locations between cycles has varied (Figure 1, upper diagonal) but shows considerable lack of connectedness over years. On average, genotypes were evaluated in 25 different locations, a value that roughly agrees with the number of locations per year. Only two genotypes were present in more than 60 locations; one is a genotype with a 25% synthetic genome resulting from the cross between CROC_1/AE.SQUARROSA (224)//OPATA M 85/3/PASTOR and the other is a genotype with a 50% synthetic genome, resulting from the cross between ALTAR 84/AEGILOPS SQUARROSA (TAUS)//OPATA M 85.

The complete data set contains 4590 observations on synthetic derived wheat lines with 50, 25, 12.5 and 6.25 percent of a synthetic genome, produced by backcrossing synthetic wheat to *T. aestivum* in successive generations, each distributed over different numbers of years and locations. The analyzed trait was grain yield (t/ha) (Table 2). Pedigree information was also available for the 88 crosses including the parental lines of the synthetics (*Ae. tauschii* and *T. turgidum*) and the bread wheat lines (*T. aestivum*) used in the crosses and backcrosses (Table 3). We used these specific experimental data to estimate the additive genetic variance among wheat populations. To this end, we followed the Bayesian hierarchical approach described above.

**Table 2:** Mean and standard deviations (SD) of grain yield (t/ha) observed in synthetic derivative crosses

		Number of			Mean	SD
Synthetic derivatives	% of Synthetic	Data points	Locations	Cycles	(t/ha)	(t/ha)
*SHW* × *V*	50	882	114	8	3.94	1.89
*V* × (*SHW* × *V*)	25	2,486	128	9	4.17	2.08
*V* × [*V* × (*V* × *SHW*)]	12.5	1,045	99	7	4.40	2.26
*V* × {*V* × [*V* × (*V* × *SHW*)]}	6.25	177	31	2	4.09	2.52
Total		4,590				

SHW, synthetic wheat; V, *T. aestivum*; % of synthetic, percent of synthetic genome. Number of data points (observations), number of locations, and number of cycles for which data were available in the SAWYT database.

**Table 3:** Number of parents and crosses in the SAWYT database

Parents	*N*	Crosses	*N*
*Ae. tauschii* (*D*)	10	(*SHW *×* V*)	12
*T. turgidum* (*T*)	21	*V* × (*SHW *×* V*)	45
*Synthetic* (*SHW *=* D *×* T*)	16	*V* × [*V* × (*V*× *SHW*)]	24
*T. aestivum* (*V*)	105	*V* × {*V* × [*V* × (*V *×* SHW)*]}	7
Total	152		88

### *Data set 2* (includes synthetic wheat lines, SHW)

This data set contains 422 synthetic wheat lines (*SHW*) lines for which *Pyrenophora tritici-repentis* (PTR) diseases were recorded. The *Pyrenophora tritici-repentis* (PTR) causes a disease originally called yellow spot but also known as tan spot, yellow leaf spot, yellow leaf blotch or helminthosporiosis. The 422 wheat lines were evaluated with six replicates in the greenhouse. The total number of observations was 438 × 6 = 2628, for which the PTR was measured. We used the same hierarchical Bayesian approach as in Data set 1.

**Figure 2:**
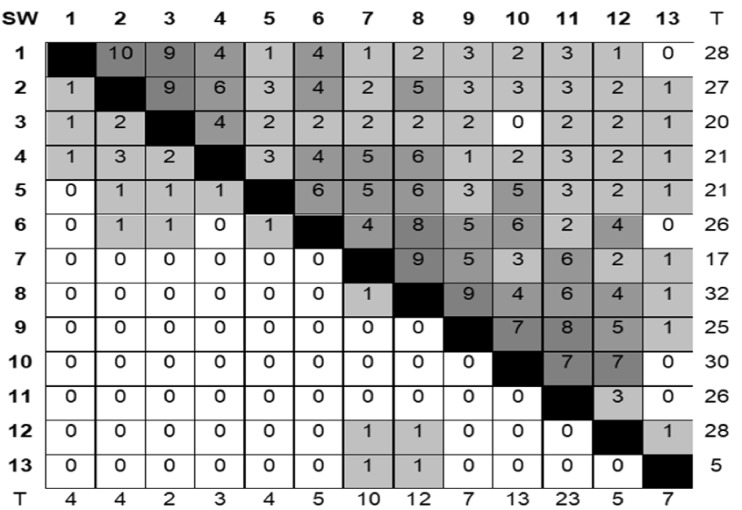
Characteristics of the synthetic derivative data file. Number of genotypes (lower diagonal) and number of locations (upper diagonal) in common across 13 cycles of CIMMYT Semi-Arid Wheat Yield Trials (SW) conducted from 1997 to 2010. The (T) refers to the total number of genotypes (columns) and locations (rows) in each SW cycle. For example, in cycle 1 there were 4 genotypes tested, of which, 1 was also tested in SW2, 1 in SW3 and 1 in SW4. There were 28 locations used in total for testing genotypes in SW1, 10 of which were also used for testing in SW2, 9 for testing in SW3, etc.

### Software for computing the numerator relationship matrices for each breed group, for model fitting, and for variance components estimation

#### FORTRAN subroutine

A FORTRAN 90 subroutine was written to compute the entries of the partial numerator relationship matrices (***A***_*Q*_) (briefly described in Appendix 3 and codes given in Supplementary File S2) associated with the additive and segregation genetic variances of each breed group (population). The algorithm for calculating the entries was developed according to the rules described by García-Cortés and Toro (2006), as described above.

#### BGLR R-package

The full conditional distributions of the model parameters [equations (A2.9)–(A2.11) from Appendix 2] are analytically recognizable and thus can be sampled using standard procedures to generate a Markov Chain by using the Gibbs sampler algorithm to generate samples from the joint posterior distribution (Wang *et al.* 1993; Jensen *et al.* 1994; Sorensen and Gianola 2002). Implementation of the Gibbs sampler involves successively sampling the vector of unknowns from the corresponding *a posteriori* conditional distribution. Once the algorithm converges, sequential sampling of conditional distributions results in sampling of the posterior marginal distributions of any parameter of interest. To estimate variance components by means of the Gibbs sampler, the model was fitted using the BGLR-R package (Pérez and de los Campos 2014), which implements a variety of shrinkage and variable selection regression procedures with high dimensional data (*e.g*., markers) and relationship matrices provided by the user (*e.g*., derived from pedigree) using the Gibbs sampling algorithm. The hyper-parameters for the prior distributions were set using the default rules implemented in the BGLR-R package (see Appendix B in the BGLR package).

Models were fitted and inferences for each fit were based on 10,000 samples which were obtained after discarding 10,000 samples that were taken as burn-in, and convergence was checked by inspecting the trace plots of variance parameters (see Supplementary File S3). Variance component estimates were represented by the average values of their posterior marginal distribution.

Additive and segregation variances of the *SHWD* breed groups were computed by weighting the BGLR estimates of additive and segregation variances of each population (*D*, *T*, and *V*) and segregation (*DV*, *TV*) by the $f_{i}^{\mathrm{BG}}$ coefficients of individual *i* defined in methods. The formulae for the variance of each breed group are summarized in Table 1. Line mean repeatability (or broad sense heritability) for each breed group was calculated as the ratio between the wheat line variance over the total phenotypic variance for the references breed group. In addition, narrow sense heritability ($H_{\mathrm{LM}}^{2}$) were estimated for each *SHWD* breed group. Narrow sense heritability was defined as the ratio between the total additive variance and the phenotypic variance for the referenced breed group (Fehr 1987; Arief *et al.* 2015). Thus,

(19) $$H_{LM}^{2}=\frac{\sigma_{A}^{2}}{\sigma_{P_{LM}}^{2}}=\frac{\sigma_{A}^{2}}{\sigma_{A}^{2}+\frac{\sigma_{GA}^{2}}{yl}+\frac{\sigma_{e}^{2}}{\mathrm{ylb}}},$$

where *y*, *l* and *b* are the average values of the number of years, locations and replications, respectively, used to evaluate genotype performance, which were calculated as follows:

(20) $$yl=\frac{n_{\mathrm{GYL}}}{n_{G}}\;b=\frac{n}{n_{\mathrm{GYL}}},$$

where *n_GYL_* is the number of genotypes by year by locations, *n*_G_ is the number of genotypes and *n* is the number of data points. We also computed the narrow sense heritability when considering all five categories in only one breed population.

#### Stan package

Additionally, in order to double check the results obtained from the BGLR, we fitted model (13) using the Stan package (Carpenter *et al.* 2017), which is a general purpose software program to fit models using the Bayesian framework. The Stan package uses the NUTS algorithm (No-U-Turn-Sampler) to obtain samples from the joint posterior distribution of the parameters of interest. We assigned the same prior distributions as in the BGLR package and the model was fitted within the R statistical package using the rstan library (Stan Development Team 2020). In order to run 10,000 iterations for fitting model (13) with the Data set 1, BGLR took ∼5 minutes using a Quad Core Intel Core i7 processor @ 2.8 GHz with 16 GB of RAM memory. On the contrary, for completing the same 10,000 iteration the Stan package took about 2.5 days in the same computer.

### Data availability

#### Supplemental files with data, software, and analytical results

Phenotypic and pedigree data, all the results, and the computer codes used for fitting the models are provided at the link https://hdl.handle.net/11529/10548407. Details of the full content of the link are given in the word file Supplementary Files S1–S3. Note that the ‘sum of the breeding values by origin’ is called ‘global’ in the three Supplementary files. A brief description of the content of each Supplemental file S1, S2 and S3 is given below.

Supplementary File S1 contains synthetic (*SHW*) (Data set 2) and synthetic derivatives’ phenotypic data (*SHWD*) (Data set 1) of the CIMMYT wheat SAWYT data sets. It includes grain yield (t/ha), environment ID, genotype ID and their proportion of the synthetic genome, and also contains pedigree files of both data sets. Supplementary File S2 contains for each data set (1) the R codes for fitting the one-breed and multiple breed models using the BGLR package, (2) the R codes for fitting the one-breed and multiple breed models using the Stan package (Stan Development Team 2020), and (3) the FORTRAN 90 subroutine to compute the partial numerator relationship ***A***_Q_ matrices briefly described in Appendix 3. It also contains several files related to numerator relationship matrices, both showing their content and describing the building process used to compute the breeding values of the wheat lines in each breed group.

Supplementary File S3 contain the results from Data set 1 using the five numerator relationship matrices, the BLUP of the breeding value of the five categories *D, T, V, DV, TV*, and the sum of the breeding values by origin (or recomposing the decomposition by breed origin). Results from Data set 2 (two breed categories) are also given. Supplementary files contain pdf and Excel files with the results for comparing BLUPs estimated using the breed categories and the two segregation populations under the two R-software programs, as well as for comparing them when considering only one-breed (homogeneous) populations. It also contains the pdf files corresponding to the trace plots and the posterior densities of each of the variance components.

## Results

### Data set 1 (synthetic wheat derivatives, *SHWD*)

Table 4 and Table A4.1 (Appendix 4) show the estimated additive genetic variances for the multi-breed or one-breed model obtained by Gibbs sampling implemented using the BGLR and Stan R packages, respectively. Both software programs provided similar results in terms of the mean additive genetic variance. Using the estimates from BGLR, the proposed model accounts for the multi-breed and one-breed genetic structures. The breed categories *D* and *T* had the largest additive genetic variance (0.616 and 0.613, respectively, from BGLR), whereas *V* had 0.182. Segregation component *TV* had larger variance (0.327) than the segregation component *DV* (0.161) (both similar for BGLR and Stan packages). The HPD95 = 95% high posterior density intervals from the Gibbs sampler implemented in the BGLR package and Stan software provided very similar lower and upper intervals. The residual variance had a variance of 0.682, whereas the genotype × environment interaction had the lowest variance (0.105 and 0.103 for BGLR and Stan, respectively). The additive genetic variance estimate for the one-breed model was 0.122 and 0.120 for BGLR and Stan, respectively.

**Table 4:** Data set 1

					HPD95	
Variance component	υ	S^2^	Mean	SD	LOWER	UPPER
Three breeds (*D, T, V*) and two segregation effects (*TV, DV*)					
* *Error	5	3.114	0.682	0.018	0.648	0.721
* *Genotype × environment	5	0.445	0.105	0.014	0.077	0.133
* Ae. tauschii* (*D*)	5	1.852	0.616	0.182	0.319	0.996
* T. turgidum* (*T*)	5	1.862	0.613	0.178	0.319	0.988
* T. aestivum* (*V*)	5	0.643	0.182	0.048	0.097	0.278
* Ae. tauschii-T. aestivum* (*DV*)	5	0.612	0.161	0.041	0.087	0.244
* T. turgidum-T. aestivum* (*TV*)	5	1.225	0.327	0.086	0.177	0.500
One-breed					
* *Error	5	3.114	0.670	0.018	0.635	0.704
* *Genotype × environment	5	1.038	0.127	0.013	0.099	0.153
* *Genotype	5	1.117	0.122	0.021	0.086	0.167

Prior degree of freedom (υ), parameter ($S^{2}$) and posterior statistics of each variance component of the model. Mean and standard deviation (SD). HPD95 = 95% high posterior density intervals from the Gibbs sampler implemented in the **BGLR R-package** for three breeds, two segregations populations and one-breed.

Table 5 shows the estimated additive genetic variances, segregation variances, and total additive genetic variances for the synthetic derivative breed groups (*SHWD*) present in the SAWYT data base (with 50, 25, 12.5 and 6.125 percent of the synthetic genome). Based on BGLR estimates of the additive genetic variance of each population *D*, *T* and *V* and on the segregation variances *DV* and *TV*, the total genetic variance for each synthetic derivative breed group was computed as the weighted sum of additive variance by source of variability. The additive variance of breed group (*SHW* × *V*) (50% of synthetic genome) was higher (0.705) compared to the other derivative breed groups with distinct proportions of synthetic genome (0.444 for 25% synthetics, 0.313 for 12.5% synthetic and 0.247 for 6.25% synthetic). On the contrary, the breed group *V* × (*V*× *SHW*) (25% of synthetic genome) had the highest segregation variance (0.408) and thus the highest total additive variance (0.852), whereas {*V*×[*V* ×[*V* × (*V*× *SHW*)]]} had low segregation variance (0.178) and the lowest total additive variance (0.425).

**Table 5:** Estimates of additive genetic variances (t/ha)^2^ for grain yield, narrow sense heritability and line mean repeatability in synthetic derivative breed groups developed by the different crosses present in the SAWYT database

Progeny	*SHW*× *V*	*V* × (*SHW* × *V*)	*V* × [*V* × (*V*× *SHW*)]	*V* × {*V* × [*V* × (*V*× *SHW*)]}
% Synthetic	50	25	12.5	6.25
Additive variance	0.705	0.444	0.313	0.247
Segregation variance	0	0.408	0.306	0.178
Total additive variance	0.705	0.852	0.619	0.425
Narrow sense heritability	0.47	0.52	0.44	0.35
Line mean repeatability	0.97	0.98	0.97	0.96

SHW, synthetic derivative, *V*, *T. aestivum*.

Narrow sense heritability ranged between 0.35 {*V*×[*V* ×[*V* × (*V*× *SHW*)]]} to 0.52 (*V*× *SHW*) and line mean repeatability varied between 0.96 to 0.98. Considering only one breed group, narrow sense heritability and line mean repeatability were 0.13 and 0.87, respectively. These last results show that partitioning the total additive variance into different breed groups should provide a more precise variance component and heritability estimations.

The BLUP prediction for each genotype breeding value and their breed-by-origin components as well as the sum of the breeding values by breed origin are given in the Excel files in Supplementary File S3. In addition, the BLUPs obtained when considering only one breed group are also included. Supplementary File S3 also contains the pdf files corresponding to the trace plots and the posterior densities of each of the variance components.

Comparing the breeding value (BLUP) estimates when considering one homogeneous group versus the sum of breeding values by breed origin gave a correlation of 0.9079, indicating similar pedigree relationships of both numerator relationship matrices. The breeding values (BLUPs) for the *SHWD* lines belonging to the *D* and *T* breed groups had a correlation of 0.6174 and 0.6211 with the corresponding BLUPs computed assuming a single additive variance, whereas the BLUPs of *V*, *DV*, and *TV* were strongly correlated: 0.8941, 0.8478, and 0.8384, respectively.

### Data set 2 (synthetic wheat, *SHW*)

In Data set 2, there are only two breed groups (*Ae. tauschii* and *T. turgidum*) (synthetics, *SHW* populations) and no segregation effects because no F_1_ and F_2_ generations were derived. This data set had the lines derived from crosses after doubling the number of chromosomes by colchicine. Table 6 and Table A4.2 (Appendix 4) show the estimated additive genetic variance for the multi-breed or one-breed model obtained by Gibbs sampling implemented using the BGLR and Stan R package, respectively. Both software packages provided similar results in terms of the mean additive genetic variance.

**Table 6:** Data set 2

					HPD95	
Variance component	υ	S^2^	Mean	SD	Lower	Upper
Two-breeds						
* *Error	5	0.445	0.417	0.008	0.400	0.435
* Ae. tauschii* (*D*)	5	0.226	0.117	0.023	0.071	0.163
* T. turgidum* (*T*)	5	0.223	0.101	0.020	0.063	0.142
One-breed						
* *Error	5	0.445	0.418	0.009	0.400	0.435
* *Genotype	5	0.453	0.235	0.019	0.197	0.273

Prior degree of freedom (υ*)*, parameter ($S^{2}$) and posterior statistics of each variance component of the model. Mean and standard deviation (SD). HPD95 = 95% high posterior density intervals from the Gibbs sampler implemented in the BGLR R-software for two breeds and one-breed.

As in the previous case, we computed the BLUPs considering each of the 2 breeds and summing up the breeding values by origin. We also computed the additive variance when considering two and one-breed populations. The two breed categories had variance components of 0.117 and 0.101 for BGLR and 0.118 and 0.1000 for Stan software. The HPD95 = 95% high posterior density intervals from the Gibbs sampler implemented in the BGLR package and Stan software provided very similar lower and upper intervals. The residual variance had similar variances of 0.418 and 0.417 for BGLR (Table 6) and Stan (Table A4.2, Appendix 4), respectively. The variance component for one-breed was high, 0.235 and 0.233 for BGLR and Stan, respectively, showing that it is redistributed when the two-breed model is fitted.

The BLUP prediction for each breeding *SHW* wheat line and their breed-by-origin components are given in the Excel files in Supplementary File S3. In addition, the BLUPs obtained when considering only one-breed group are also included. Supplementary File S3 also contains the pdf files corresponding to the trace plots and the posterior densities of each of the variance components. The correlations between the prediction of the breeding values (BLUPs) for the two categories, and the sum of the breeding value by origin for the 422 lines between BGLR and Stan were high. Comparing the breeding value (BLUP) estimates when considering one-breed versus the recomposing breeding values obtained by fitting the multi-breed model gave a correlation of around 0.90.

## Discussion

In the current research, we implemented a Markov Chain Monte Carlo (MCMC) procedure to estimate the additive genetic variance for synthetic wheat crosses with a hierarchical Bayesian model. The model we fitted involved a genetic structure based on the population from which the alleles that make up the cross originated (Elzo and Bradford 1985; Elzo 1990; Wei *et al.* 1991). Because the genome of the synthetic originates in different populations and its ploidy agrees with those of the base populations (1/3: 2/3), the breeding value of their progeny must also be calculated on the basis of this ploidy. This is also true for the hexaploid *T. aestivum* population included in the crosses that produced the synthetic derivative lines. Therefore, it was necessary to consider a genetic model that partitions the synthetic derivative breeding value into components that can be attributed to the different genetic sources. Thus, we adapted the theory developed for multi-breed animal populations (Lo *et al.* 1993; Cantet and Fernando 1995; García-Cortés and Toro 2006; Munilla-Leguizamón and Cantet 2010) to species with variable parental ploidy levels, such as synthetic wheat. The proposed model was adjusted according to a database of multi-environment experimental data on synthetic wheat and their derivatives, which contains historic observations of the grain yield of a considerable number of genotypes and locations.

The standard model with a single variance component was also fitted using the BGLR and Stan software packages. The hyper-parameters for the prior distributions were set according to the internal rules implemented in the BGLR package which assigns weakly informative priors. In the case of Data set 1, the linear mixed model included fixed environmental effects, the genotype × environment interaction, and the breeding value, as a random effect, with a covariance structure based on the relationship between *SHWD* lines. In the case of Data set 2, the model included only the breeding value of the *SHW* lines. In both cases, relationship matrices were derived from the corresponding pedigree.

### Partitioning additive genetic variance for different breeds

Partitioning the genotypic variability for Data set 1 revealed that the three wheat species had different additive genetic variances for grain yield, with *T. aestivum* (0.182) showing less genetic variability than *T. turgidum* (0.613) and *Ae. tauschii* (0.616). This finding agrees with the molecular results for genetic diversity of Warburton *et al.* (2006), Dreisigacker *et al.* (2008), Lage *et al.* (2003) and Zhang *et al.* (2005), in the sense that the variability is lower than the one from *Ae. tauschii* and its durum parents, even when compared with the variability present in the derived synthetic lines (Jafarzadeh *et al.* 2016). The fact that the estimated segregation variances were, within the 95% HPD interval, greater than zero indicates differences in the allelic frequencies (Wright 1968; Lande 1981; Birchmeier *et al.* 2002) among the *T. turgidum-T. aestivum* (0.327) and *Ae. tauschii-T. aestivum* populations (0.161).

For Data set 1, the segregation variances estimated by the model were, within the 95% HPD interval, greater than zero (Table 4) and (Table 4.1, Appendix 4). They represent an additional source of variability of the genetic variance that can be attributed to differences in allelic frequencies between the populations (Wright 1968; Lande 1981; Birchmeier *et al.* 2002). The difference between the estimates of segregation variances of the *T. turgidum—T. aestivum* and *Ae. tauschii- T. aestivum* populations (0.327 *vs* 0.161) suggests that the differences in the allelic frequencies between *T. turgidum—T. aestivum* populations are higher than those between *Ae. tauschii- T. aestivum* populations*.* It is difficult to determine what the causes of these differences are because they may be related to genetic drift, to selection, or to a combination of both (Falconer and Mackay 1996).

For Data set 2, which combined only the cross between *T. turgidum* and *Ae. tauschii*, both had similar contributions (0.117 and 0.101) and different when considering one-breed group (0.235) for BGLR and *T. turgidum =* 0.100 and *Ae. tauschii* = 0.118, and 0.235 when considering one-breed group for BGLR and 0.233 from Stan software.

Given that the genetic variances of wheat populations may be different, it is relevant to take these differences into account while predicting the breeding values of synthetic lines and their parents. BLUP methodology is essentially a shrinking technique that requires modeling the genotypic means while accounting for the covariance among genetic effects. Under this methodology, breeding values are modeled as Gaussian distributed random variables with a covariance matrix that results from the product of a genetic relationship matrix, ***A***, and an additive genetic variance parameter, $\sigma_{A}^{2}$. Differences in the additive variance parameter affect the level of shrinkage. The greater the genetic variance of a trait, the lower the shrinkage (Xavier *et al.* 2016) assuming similar error variances. On the contrary, Henderson (1975) showed that using a wrong covariance matrix leads to predictors that, although unbiased, are not the “best,” *i.e*., they are not “minimum variance.” Therefore, incorrectly specifying the variance parameters will have a negative impact on the estimation of the realized breeding values and prediction errors. This study showed the advantage of using a multi-breed model for assessing the additive variance in the different breeds and planning wheat breeding strategies accordingly. Although in this study we assumed a fully additive model, it should be noted that an important amount of genetic variance in wheat is epistatic.

Of all unbiased linear predictors of genetic effects, BLUPs are the ones that have the lowest prediction errors under certain assumptions and with known dispersion parameters (Searle *et al.* 1992). According to Piepho *et al.* (2008), even when the dispersion parameters are estimated along with the prediction, and considering that some assumptions may not hold (for example, that the population is under selection by means of nonrandom crossing systems), the “empirical” BLUP predictors (EBLUP) are robust and will not be very far from the prediction error variance of the data generating process. This is especially true if the base population is included in the analysis, and the covariance structure of the genetic effects is determined by the relationships among all genotypes used for selection purposes (Crossa *et al.* 2006; Piepho *et al.* 2008; Kelly *et al.* 2009).

Several authors have indicated the methodological advantages of including the data generated during different selection cycles in one meta-analysis in which the BLUPs could be used to predict the breeding values of all the genotypes simultaneously, rather than to evaluate sub-groups of genotypes that are the result of intense selection in previous cycles and then assume that the effects of those cycles are independent (Kelly *et al.* 2009; Kerr *et al.* 2012; Arief *et al.* 2015). Estimated breeding values obtained in the latter manner are not expressed on an adequate population scale due to the absence of base population individuals that were discarded in previous cycles and of the implicit genetic association between them (Bauer *et al.* 2006). In contrast, a model such as the one fitted here allows evaluating the history of the materials used in selection including several improvement cycles, from the base population to the most recent progeny. It should be noted here that systematically ignoring the relationships among genotypes, especially in unbalanced designs, tends to result in the selection of the “oldest” genotypes for which there are large numbers of observations. In contrast, incorporating genetic relationships based on either pedigree or molecular information will make it possible to detect promising parent lines that will produce new genotypes, even if there are very few observations of those lines (Bauer *et al.* 2006).

### The hierarchical Bayesian model

Under the Bayesian paradigm, uncertainty in the values of the variance components, as well as of any other parameter, is dealt with by probability distributions describing such uncertainty. In the hierarchical model developed here, variance components were assumed to follow, *a priori*, a scaled inverse Chi-square distribution. After applying the Bayes theorem, these priors were combined with the likelihood function and conjugated full conditional posterior distributions were obtained. Finally, inference was accomplished by systematically sampling from these conditional distributions (Wang *et al.* 1993). Although we did not use them in this study, REML estimators can also be used for estimating additive genetic variances (Crossa *et al.* 2006; Kelly *et al.* 2009) and even segregation variances (Birchmeier *et al.* 2002). However, in general, the Bayesian approach is more intuitive and flexible, and the results are more informative as compared to those of maximum likelihood methods (Munilla-Leguizamón and Cantet 2010). For example, confidence intervals and even the full marginal distribution are readily available from the MCMC samples, although the method is computationally demanding, especially for a highly parametrized model such as the one implemented here. Samples from the posterior distributions can be obtained using different algorithms; in the case of the BGLR package (Pérez and de los Campos 2014), the samples are obtained using a standard Gibbs sampler, whereas in the case of the Stan package, the samples are obtained using the NUTS algorithm.

### Analyses of the data sets

We analyzed two distinct data sets in the present research. The first one is the historical SAWYT data set with different numbers of breed groups (Data set 1 contains *SD*). The other one, with a simpler structure, includes only synthetics (Data set 2 with only *S*). The joint analysis of the SAWYT database that contains historic observations on the grain yield of a considerable number of genotypes and locations produced estimates of the seven variance components of the model (Tables 4 and 5). The most relevant estimates were the ones associated with the additive genetic variances of the three pure wheat populations involved in the crosses of the evaluated genotypes: ${\hat{\sigma }}_{A,D}^{2}$, ${\hat{\sigma }}_{A,T}^{2}$, and ${\hat{\sigma }}_{A,V}^{2}$. Using these values, it is possible to estimate the additive variances of the different groups of derived synthetic lines, based on the proportion of the genome of the pure *D*, *T* and *V* populations present in the crosses, plus the segregation variance (Table 6). The magnitude of the estimated additive variance was systematically reduced in the crosses with a proportional decrease in the synthetic genome, and this is surely associated with the fact that the variance of the *T. aestivum* population was the smallest. Therefore, as the proportion of synthetic genome declines in successive backcrosses, the genetic variability available for breeding decreases.

The genetic parameters estimated for Data set 1 in the current research were higher than the values reported in the literature for the genotypic variance of wheat estimated using different databases. Dreccer *et al.* (2007) used data of synthetic derivative wheat lines from three different groups of multi-environment trials in Mexico and Australia and estimated additive genetic variances equal to 0.018, 0.048 and 0.037. In turn, the estimates of Rattey and Shorter (2010) for the genetic variance of conventional hexaploid lines (*T. aestivum*) and *derived* synthetic CIMMYT lines (grown in trials under drought in subtropical environments in Mexico and Australia) was 0.0121. Arief *et al.* (2015) found a value of 0.04 for the genotypic variance of the grain yield of 900 wheat lines included in an extensive CIMMYT database called “Elite Spring Wheat Yield Trials” (ESWYT), which is characterized by trials located in irrigated areas with high yield potential (Trethowan *et al.* 2003).

### Implication of variance component estimations for wheat breeding

The result of modeling the genetic effects of wheat lines as random variables has several implications for crop improvement. In the first place, it makes it possible to predict the breeding value of the *Ae. tauschii* and *T. turgidum* parents and the hybrids produced by crossing, and the synthetic lines, for which there are no phenotypic observations. In addition, only by evaluating the segregating populations of the crosses of synthetic lines with *T. aestivum* can new and positive alleles be identified to improve grain yield in wheat (Jafarzadeh *et al.* 2016). It is also possible to predict breeding values of the synthetic derivative lines, which can be selected based on their general performance or by the breeding value component associated with one of the populations from which they originated. Secondly, it provides an estimation of the genetic variability (additive variances) that can be compared with the phenotypic variability, making it possible to estimate the heritability of the trait. In this sense, grain yield total additive variance and heritability based on Data set 1 was higher when synthetic derivatives crosses were analyzed considering the corresponding breed groups proposed here, relative to the one-breed population model.

Genotype × environment interaction (G × E) has been identified as being a very important component of the genotypic variability of grain yield of several crops (Crossa *et al.* 2006; de la Vega *et al.* 2007; DeLacy *et al.* 2010). The variance component contributed about 10 to 25% of total variability (Lecomte *et al.* 2010) and therefore must be included in genetic evaluation models. It was included here because it is useful for quantifying these effects and differentiating the adaptive patterns of the genotypes in the different environments. It also reduces the residual variability, as well as the error of prediction of the breeding values. For Data set 1, the (G × E) variance component was estimated to be 0.105, a smaller value than the ones estimated in other trials: (0.034–0.187, Dreccer *et al.* 2007; 0.238, Cooper *et al.* 2001; 0.239, Arief *et al.* 2015 ). It is important to recall that one of the goals of this research was to estimate the overall performance of the genotypes, but not to estimate the positive or negative effects of the performance in the different environments precisely. This would require fitting a more complex statistical model, for example, one that includes the factor analytic model for the interaction effects from the genotypic correlations matrix (Crossa *et al.* 2006; Kelly *et al.* 2009).

It is possible to treat a character in different environments as different traits and fit a multi-trait BLUP using correlations between environments. The factor analytic model could provide main environmental causes of G × E, only if factors and loadings are associated with some environmental variables like soil type, moisture availability, altitude, frost prevalence, precipitations, sun radiation, etc. However, the lack of connectedness between locations across years of these experimental data makes it unsuitable to attempt a more complex structure for the G × E effects. This would be possible with additional environmental information about the locations and years in order to model the covariance between environments.

## Conclusions

Estimates of the genetic variance parameters, obtained under a hierarchical Bayesian approach, evidenced that genetic variability is heterogeneous among wheat populations. The variances of *T. turgidum* and *Ae. tauschii* were the greatest, whereas the additive variance of *T. aestivum* was less than half the size of the previous ones, a result that is consistent with previous reports pointing toward less genetic variability in commercial elite wheat varieties. These results are also evidence that synthetic hexaploid wheat is helpful for increasing the genetic diversity of cultivated bread wheat. The analyses of two different data sets comprising either synthetic derivatives of wheat or only synthetic wheat proved the suitability of the statistical model used in this study for accounting for complex genetic variance-covariance structures existing in multi-breed populations and their segregation populations, including breed groups with different ploidy levels. The model used in this study should offer useful results for wheat genetic resource conservation when making wide crosses, establishing correct pre-breeding strategies, and determining efficient wheat breeding schemes for accelerating genetic gains.

## Appendix 1

### Synthetic F_1_ derivatives

We we will first obtain the variance of a cross between a synthetic line and a genotype belonging to *T. aestivum* which would give rise to an *F*_1_  *synthetic* derivative line. Using (1), an additive genotypic model for this cross is represented by:

(A1.1) $$G_{i}^{SD}=\mu +\alpha_{P_{i1}}^{D}+\alpha_{P_{i1}}^{T}+\alpha_{P_{i2}}^{T}+\alpha_{M_{i1}}^{V}+\alpha_{M_{i2}}^{V}+\alpha_{M_{i3}}^{V}$$

Expression (A1.1) results from the fact that the hexaploid *F*_1_  *synthetic* derivative line inherits three alleles from the male parent (a synthetic line) and three alleles from a female parent (*T. aestivum=V*) breed group. We further assume that the first allele inherited from the synthetic line comes from diploid *Ae. tauschii* (*D*), and the second and third alleles come from tetraploid *T. turgidum* (*T*).

Now, taking variance to expression (A1):

(A1.2) $$\begin{array}{c} \text{Var}\left(G_{i}^{SD}\right)=\text{Var}\left(\mu +\alpha_{P_{i1}}^{D}+\alpha_{P_{i1}}^{T}+\alpha_{P_{i2}}^{T}+\alpha_{M_{i1}}^{V}+\alpha_{M_{i2}}^{V}+\alpha_{M_{i3}}^{V}\right)\\ =\text{Var}\left(\alpha_{P_{i1}}^{D}\right)+\text{Var}\left(\alpha_{P_{i1}}^{T}\right)+\text{Var}\left(\alpha_{P_{i2}}^{T}\right)+2\text{Cov}\left(\alpha_{P_{i1}}^{T},\alpha_{P_{i2}}^{T}\right)+\\ +\text{Var}\left(\alpha_{M_{i1}}^{V}\right)+\text{Var}\left(\alpha_{M_{i2}}^{V}\right)+\text{Var}\left(\alpha_{M_{i3}}^{V}\right)+2\text{Cov}\left(\alpha_{M_{i1}}^{V},\alpha_{M_{i2}}^{V}\right)+\\ +2\text{Cov}\left(\alpha_{M_{i1}}^{V},\alpha_{M_{i3}}^{V}\right)+2\text{Cov}\left(\alpha_{M_{i2}}^{V},\alpha_{M_{i3}}^{V}\right) \end{array}.$$

As before, all covariances among effects of purebred alleles were assumed to be zero in (A1.2). Although *T. turgidum* and *T. aestivum* are allopolyploid, all covariance among alleles from distinct genomes ($\alpha_{P_{\mathrm{i1}}}^{T}$ and $\alpha_{P_{\mathrm{i2}}}^{T}$; $\alpha_{D_{\mathrm{i1}}}^{V}$ and $\alpha_{D_{\mathrm{i2}}}^{V}$; etc.) have very low probability of being IBD; therefore, they are assumed independent, and covariances in (A1.2) are equal to zero. Now, by applying similar considerations as in the case of the synthetic *Ae. tauschii* × *T. turgidum* line, the expression can be condensed further to:

(A1.3) $$\begin{array}{c} \text{Var}\left(G_{i}^{SD}\right)=\text{Var}\left(\alpha_{P_{i1}}^{D}\right)+\text{Var}\left(\alpha_{P_{i1}}^{T}\right)+\text{Var}\left(\alpha_{P_{i2}}^{T}\right)+\\ +\text{Var}\left(\alpha_{M_{i1}}^{V}\right)+\text{Var}\left(\alpha_{M_{i2}}^{V}\right)+\text{Var}\left(\alpha_{M_{i3}}^{V}\right)\\ =\text{Var}\left(\alpha_{P_{i}}^{D}\right)+2\text{Var}\left(\alpha_{P_{i}}^{T}\right)+3\text{Var}\left(\alpha_{M_{i}}^{V}\right)\\ =\frac{1}{2}\sigma_{A,D}^{2}+\frac{1}{2}\sigma_{A,T}^{2}+\frac{1}{2}\sigma_{A,V}^{2} \end{array}.$$

#### Backcross to *T. aestivum*

Finally, consider the genotypic variance of a *B_1_* line produced by backcrossing the *F*_1_ synthetic derivative breed group (male parent) to *T. aestivum* (female parent). The additive genotypic value is represented by:

(A1.4) $$G_{i}^{B_{1}}=\mu +\alpha_{P_{i1}}^{SD}+\alpha_{P_{i2}}^{SD}+\alpha_{P_{i3}}^{SD}+\alpha_{M_{i1}}^{V}+\alpha_{M_{i2}}^{V}+\alpha_{M_{i3}}^{V}.$$

The gametes segregated by the *F*_1_ synthetic derivative contain a combination of alleles with the following additive effects: 1) $\alpha_{P_{i1}}$: originated in *Ae. tauschii (D)* or *T. aestivum* (*V*), and 2) $\alpha_{P_{2i}}$ and $\alpha_{P_{3i}}$ originated either in *T. turgidum* (*T*) or *T. aestivum* (*V*). The origin of the genes is denoted by a superscript.

At this point, we introduce the formulae by Lo *et al.* (1993) to derive the genotypic variance of this breed group. As explained at the beginning of Models and Methods section, these authors expressed the genotypic variance of any crossbred group as the sum of the variance of the alleles in the purebreds, each multiplied by the respective probability of the alleles coming from this purebred group (denoted by *f* coefficients) plus segregation variances. In the case under consideration, if we substitute equations (19) and (20) of Lo *et al.* (1993) in the expression of the variance of (A1.4) and exclude null terms with zero *f* coefficients, the genotypic additive variance can be expressed as:

(A1.5) $$\begin{array}{c} \mathrm{Var}\left(G_{i}^{B_{1}}\right)=f_{P}^{D}\mathrm{Var}\left(\alpha_{P_{i1}}^{D}\right)+f_{P}^{V}\mathrm{Var}\left(\alpha_{P_{i1}}^{V}\right)+f_{P}^{D}f_{P}^{V}{\left(\varepsilon_{D}-\varepsilon_{V}\right)}^{2}+\\ +f_{P}^{T}\mathrm{Var}\left(\alpha_{P_{i2}}^{T}\right)+f_{P}^{V}\mathrm{Var}\left(\alpha_{P_{i2}}^{V}\right)+f_{P}^{T}f_{P}^{V}{\left(\varepsilon_{T}-\varepsilon_{V}\right)}^{2}+\\ +f_{P}^{T}\mathrm{Var}\left(\alpha_{P_{i3}}^{T}\right)+f_{P}^{V}\mathrm{Var}\left(\alpha_{P_{i3}}^{V}\right)+f_{P}^{T}f_{P}^{V}{\left(\varepsilon_{T}-\varepsilon_{V}\right)}^{2}+\\ +3f_{M}^{V}\mathrm{Var}\left(\alpha_{M}^{V}{}_{{}_{i1}}\right)+\frac{1}{2}\mathrm{Cov}\left(G_{P_{i}},G_{M_{i}}\right) \end{array},$$

where $\varepsilon_{BG}$ is the conditional mean of an additive effect $\alpha_{P_{i}}^{BG}$, given that the paternal allele is inherited from pure breed *BG*. In turn, the last term stands in for the covariance between the genotypic values of the parents. In (A1.5), the probability that any allele of the male parent (*P*) originated in a given pure breed group (*BG* = {*D*, *T*, or *V*}) is $f_{P}^{\mathrm{BG}}=\frac{1}{2}$. In turn, all maternal alleles derive from *T. aestivum* and thus $f_{M}^{V}=1$. Hence expression (A1.5) can be written as:

(A1.6) $$\begin{array}{c} \mathrm{Var}\left(G_{i}^{B_{1}}\right)=\frac{1}{2}\mathrm{Va}r_{D}\left(\alpha_{P_{i1}}^{D}\right)+\frac{1}{2}\;\mathrm{Va}r_{V}\left(\alpha_{P_{i1}}^{V}\right)+\frac{1}{2}\frac{1}{2}\;{\left(\varepsilon_{D}-\varepsilon_{V}\right)}^{2}+\\ +\frac{1}{2}\;\mathrm{Va}r_{T}\left(\alpha_{P_{i2}}^{T}\right)+\frac{1}{2}\mathrm{Va}r_{V}\left(\alpha_{P_{i2}}^{V}\right)+\frac{1}{2}\frac{1}{2}{\left(\varepsilon_{T}-\varepsilon_{V}\right)}^{2}+\\ +\frac{1}{2}\;\mathrm{Va}r_{T}\left(\alpha_{P_{i3}}^{T}\right)+\frac{1}{2}\mathrm{Va}r_{V}\left(\alpha_{P_{i3}}^{V}\right)+\frac{1}{2}\frac{1}{2}{\left(\varepsilon_{T}-\varepsilon_{V}\right)}^{2}+\\ +3\mathrm{Va}r_{V}\left(\alpha_{M_{i1}}^{V}\right)+\frac{1}{2}\;\mathrm{Cov}\left(G_{P_{i}},G_{M_{i}}\right) \end{array}.$$

Segregation variances are defined as in Wright (1968) and Lande (1981), *i.e*., as half the squared differences in the conditional means of the additive effects of the two breed groups involved in the segregation:

(A1.7) $$\sigma_{S_{DV}}^{2}=\frac{1}{2}\;{\left(\varepsilon_{D}-\varepsilon_{V}\right)}^{2}\;\mathrm{and}\;\sigma_{S_{TV}}^{2}=\frac{1}{2}\;{\left(\varepsilon_{T}-\varepsilon_{V}\right)}^{2}.$$

By substituting with the variances from each breed group and rearranging, one obtains:

(A1.8) $$\mathrm{Var}\left(G_{i}^{B_{1}}\right)=\frac{1}{4}\sigma_{A,D}^{2}+\frac{1}{4}\sigma_{A,T}^{2}+\frac{3}{4}\sigma_{A,V}^{2}+\frac{1}{2}\sigma_{S_{DV}}^{2}+\sigma_{S_{TV}}^{2}+\frac{1}{2}\mathrm{Cov}\left(G_{P_{i}},G_{M_{i}}\right).$$

For successive backcrosses, we can define the $f_{i}^{\mathrm{BG}}$coefficients of an offspring *i* as the average of the breed composition in the paternal and maternal breed groups, *e.g*., $f_{i}^{V}=\frac{1}{2}\left(f_{P_{i}}^{V}+f_{M_{i}}^{V}\right)$. By doing so, equation (A1.8) can be written more generally as:

(A1.9) $$\begin{array}{c} \mathrm{Var}\left(G_{i}^{SD}\right)=f_{i}^{D}\sigma_{A,D}^{2}+f_{i}^{T}\sigma_{A,T}^{2}+f_{i}^{V}\sigma_{A,V}^{2}+\\ +2\;f_{P_{i}}^{D}f_{P_{i}}^{V}\sigma_{S_{DV}}^{2}+4\;f_{P_{i}}^{T}f_{P_{i}}^{V}\sigma_{S_{TV}}^{2}+\frac{1}{2}\;\mathrm{Cov}\left(G_{P_{i}},G_{M_{i}}\right) \end{array}.$$

Expression (A1.9) allows computing the additive variance of genotypes belonging to any synthetic derivative breed group and is structurally similar to the formula for computing the additive variance of individual *i* in a multibreed population obtained by Lo *et al.* [1993, equation (31)] in the diploid case. All these rules are summarized in Table 1.

## Appendix 2

At the next hierarchical level, Scaled Inverse Chi-squared distributions were assigned as priors for the dispersion parameters, *i.e*., scalars $\sigma_{A,Q}^{2}$, $\sigma_{GE}^{2}$, and $\sigma_{e}^{2}$. Explicitly, their density functions were, respectively,

(A2.1) $$p\left(\sigma_{\mathrm{A,Q}}^{2}\;|\;\upsilon_{Q},\;S_{\mathrm{A,Q}}^{2}\right)\propto \;{\left(\sigma_{\mathrm{A,Q}}^{2}\right)}^{-\left(\frac{\upsilon_{Q}}{2}+1\right)}\;\;\text{exp}\;\;\left\{-\frac{\upsilon_{Q}\;S_{\mathrm{A,Q}}^{2}}{2\sigma_{\mathrm{A,Q}}^{2}}\right\}$$

(A2.2) $$p\left(\sigma_{\mathrm{GE}}^{2}\;|\;\upsilon_{\mathrm{GE}},\;S_{\mathrm{GE}}^{2}\right)\propto \;{\left(\sigma_{\mathrm{GE}}^{2}\right)}^{-\left(\frac{\upsilon_{\mathrm{GE}}}{2}+1\right)}\;\;\text{exp}\;\;\left\{-\frac{\upsilon_{\mathrm{GE}}\;S_{\mathrm{GE}}^{2}}{2\sigma_{\mathrm{GE}}^{2}}\right\}$$

(A2.3) $$p\left(\sigma_{e}^{2}\;|\;\upsilon_{e},\;S_{e}^{2}\right)\propto \;{\left(\sigma_{e}^{2}\right)}^{-\left(\frac{\upsilon_{e}}{2}+n+1\right)}\;\;\text{exp}\;\;\left\{-\frac{\upsilon_{e}\;S_{e}^{2}}{2\sigma_{e}^{2}}\right\},$$

where S^2^ represent scale hyperparameters that should be interpreted as statements about the expectation of the prior distributions. In turn, υ are the “hyper” degrees of freedom, and are interpreted as prior degrees of belief. Both sets of values are defined by the analyst.

To specify the joint posterior distribution, we assumed that **b**, ***a****_Q_* |$\sigma_{A,Q}^{2}$, *Q =* {*D, T, V, DV, TV*}, $\mathrm{ge}|\sigma_{GE}^{2}\;\mathrm{and}\;\sigma_{e}^{2}$ are all independent *a priori*. Thus, the joint posterior distribution will be proportional to the product of the likelihood function times each prior density, as follows:

(A2.4) $$\begin{array}{c} p\left(b\;,\;a_{Q},\mathrm{ge},\sigma_{\mathrm{A,Q}}^{2},\sigma_{\mathrm{GE}}^{2},\sigma_{e}^{2}\;|\;y\right)\;\\ \;\propto p\left(y\;|\;b\;,\;a_{Q},\mathrm{ge},\sigma_{e}^{2}\right)\times p\;\left(b\;|\;K\;\right)\times \\ \;\times \prod_{Q}p\left(a_{Q}|A_{Q},\sigma_{\mathrm{A,Q}}^{2}\;\right)\times \prod_{Q}p\;\left(\sigma_{\mathrm{A,Q}}^{2}\;|\;S_{\mathrm{A,Q}}^{2},\upsilon_{Q}\right)\times \\ \;\times p\left(\mathrm{ge}|\sigma_{\mathrm{GE}}^{2}\;\right)\times p\left(\sigma_{\mathrm{GE}}^{2}\;|\;S_{\mathrm{GE}}^{2},\upsilon_{\mathrm{GE}}\right)\times p\left(\sigma_{e}^{2}|S_{e}^{2},\upsilon_{e}\right) \end{array}.$$

By replacing the kernels of the density functions and after grouping the common factors together, we obtain:

(A2.5) $$\begin{array}{c} p\left(b\;,\;a_{Q},\mathrm{ge},\sigma_{\mathrm{A,Q}}^{2},\sigma_{\mathrm{GE}}^{2},\sigma_{e}^{2}\;|\;y\right)\;\\ \;\propto \;\text{exp}\;\;\left\{-\frac{1}{2}\;b^{\prime }K^{-1}b\right\}\;\\ \;\times \prod_{Q}\left(\text{exp}\;\left\{-\frac{a_{Q}^{\prime }\;A_{Q}^{-\;1}\;a_{Q}\;+\;\upsilon_{Q}\;S_{Q}^{2}}{2\sigma_{\mathrm{A,Q}}^{2}}\right\}{\left(\sigma_{\mathrm{A,Q}}^{2}\right)}^{-\frac{1}{2}\left(\upsilon_{Q}+q_{Q}+5\right)}\;\right)\;\times \\ \;\times \exp\left\{-\frac{ge^{\prime }\mathrm{ge}+\upsilon_{\mathrm{GE}}\;S_{\mathrm{GE}}^{2}}{2\sigma_{\mathrm{GE}}^{2}}\right\}\;{\left(\sigma_{\mathrm{GE}}^{2}\right)}^{-\frac{1}{2}\left(\upsilon_{Q}+d+2\right)}\times \\ \;\times {\left(\sigma_{e}^{2}\right)}^{-\frac{1}{2}\left(\upsilon_{e}+n+2\right)}\exp\;\left\{-\frac{e^{\prime }e+\upsilon_{e}S_{e}^{2}}{2\sigma_{e}^{2}}\right\} \end{array},$$

where the random error vector***e*** is:

(A2.6) $$e=y-\mathrm{Xb}-\sum_{Q}Z_{Q}a_{Q}-Z_{\mathrm{GE}}\mathrm{ge}.$$

From expression (A2.5), it is possible to obtain the kernel of the full conditional posterior density of any parameter of interest by keeping the remaining ones fixed.

We will first describe the analytic expression of the location parameters whose joint conditional distribution is as follows:

(A2.7) $$\begin{array}{c} p\left(b\;,\;a_{Q},\mathrm{ge}\;|\;y,\sigma_{\mathrm{A,Q}}^{2},\sigma_{\mathrm{GE}}^{2},\sigma_{e}^{2}\right)\;\\ \;\propto \;\exp\;\left\{-\frac{e^{\prime }e}{2\sigma_{e}^{2}}\right\}\times \text{exp}\;\;\left\{-\frac{1}{2}\;b^{\prime }K^{-1}b\right\}\times \\ \;\times \text{exp}\;\left\{-\frac{{a^{\prime }}_{Q}\;\left[A_{Q}^{-\;1}{\left(\sigma_{\mathrm{A,Q}}^{2}\right)}^{-1}\right]\;a_{Q}}{2\sigma_{e}^{2}}\right\}\times \exp\left\{-\frac{ge^{\prime }\mathrm{ge}}{2\sigma_{\mathrm{GE}}^{2}}\right\}\; \end{array}.$$

Now, by performing the necessary algebraic operations (Jensen *et al.* 1994; Sorensen and Gianola 2002), it can be shown that:

(A2.8) $$p\left(b\;,\;a_{Q},\mathrm{ge}\;|\;y,\sigma_{\mathrm{A,Q}}^{2},\sigma_{\mathrm{GE}}^{2},\sigma_{e}^{2}\right)∼N\left({\left[\hat{b}\;,\;\;{\hat{a}}_{Q},\;\hat{g}e\right]}^{\prime },\;\;C^{-1}\sigma_{e}^{2}\;\right).$$

The expression ${\left[\hat{b}\;,\;\;{\hat{a}}_{Q},\;\hat{g}e\right]}^{\prime }=C^{-1}x$ is the solution of the mixed model equations (MME) of model (13), **C^−^**^1^ being the inverse of the coefficients matrix and ***x*** the right-hand side vector of the MME.

Next, it can be shown that the full conditional distribution of the error variance is proportional to:

(A2.9) $$p\;\left(\sigma_{e}^{2}|\;b,a_{Q},ge,\sigma_{A,Q}^{2},\sigma_{GE}^{2},y\right)\propto {\left(\sigma_{e}^{2}\right)}^{-\frac{1}{2}\left({\tilde{\upsilon }}_{e}|+2\right)}\;\text{exp}\;\left\{-\frac{{\tilde{\upsilon }}_{e}{\tilde{S}}_{e}^{2}}{2\sigma_{e}^{2}}\right\}.$$

In expression (A2.9), we can see the kernel of an inverted Chi-square density function scaled with hyperparameters ${\tilde{\upsilon }}_{e}$ and ${\tilde{S}}_{e}^{2}$.

In turn, the full conditional posterior distribution of each additive genetic variance component by source of origin *Q* (*Q* = {*D, T, V, DV, TV*}) is proportional to:

(A2.10) $$p\;\left(\sigma_{A,Q}^{2}|\;b,a_{Q},ge,\sigma_{A,P}^{2},\sigma_{GE}^{2},\upsilon_{Q},S_{Q}^{2},y\right)\propto {\left(\sigma_{A,Q}^{2}\right)}^{-\frac{1}{2}\left(q_{Q}+\upsilon_{Q}+1\right)}\exp\left\{-\frac{{\tilde{\upsilon }}_{Q}{\tilde{S}}_{Q}^{2}}{2\sigma_{A,Q}^{2}}\right\},$$

with ${\tilde{S}}_{Q}^{2}=\frac{a_{Q}^{\prime }A_{Q}^{-1}a_{Q}\;+\;\upsilon_{Q}S_{Q}^{2}}{\upsilon_{Q}}$ and ${\tilde{\upsilon }}_{Q}=q+\;\upsilon_{Q}$. In (A2.10), the symbol $\sigma_{\mathrm{A,P}}^{2}$ is used to represent the variance components of the remaining genetic additive effects taken as constants.

Next, we present the full conditional posterior distributions of the variance of the genotype by environment interaction random effect. This distribution is proportional to:

(A2.11) $$p\;\left(\sigma_{GE}^{2}|b,a_{Q},ge,\sigma_{A,Q}^{2},\sigma_{e}^{2},\;y\right)\propto {\left(\sigma_{GE}^{2}\right)}^{-\frac{1}{2}\left(\upsilon_{GE}+2\right)}\exp\left\{-\frac{{\tilde{\upsilon }}_{GE}{\tilde{S}}_{GE}^{2}}{2\sigma_{GE}^{2}}\right\},$$

where ${\tilde{\upsilon }}_{GE}=\upsilon_{GE}+d$ and ${\tilde{S}}_{GE}^{2}=\frac{\;{\mathrm{ge}}^{\prime }ge\;+\;\upsilon_{GE}S_{GE}^{2}}{\upsilon_{GE}}$. Expression (A2.11) corresponds to the kernel of a scaled inverted Chi-square distribution with parameters ${\tilde{\upsilon }}_{GE}$ and ${\tilde{S}}_{GE}^{2}$.

## Appendix 3

A FORTRAN 90 code was developed to compute the entries in the partial numerator relationship matrices (***A***_*Q*_), associated with the additive and segregation genetic variances using Lo *et al.* (1993) rules. For the construction of each ***A***_*Q*_, the code requires, for each individual, the identification of the parents (male and female parents) and the proportion of the pure breed genome *D*, *T* and *V*. The proportions are represented by the $f_{i}^{\mathrm{BG}}$coefficients described above. With these coefficients, the program assigned diagonal entries of each individual and the off diagonals are computed recursively using (12). The FORTRAN 90 codes as well as the five numerator relationship matrices are given in Supplementary File S3.

## Appendix 4

**Table A4.1:** Data set 1

					HPD95	
Variance component	υ	S^2^	Mean	SD	LOWER	UPPER
Three breeds (*D, T, V*) and two segregation effects (*TV, DV*)						
Error	5	3.114	0.687	0.020	0.646	0.721
Genotype × environment	5	0.445	0.103	0.015	0.078	0.134
*Ae. tauschii* (*D*)	5	1.852	0.603	0.183	0.306	0.963
*T. turgidum* (*T*)	5	1.862	0.615	0.177	0.319	0.953
*T. aestivum* (*V*)	5	0.643	0.184	0.050	0.105	0.281
*Ae.tauschii-T. aestivum (DV)*	5	0.612	0.161	0.038	0.083	0.228
*T. turgidum-T. aestivum*(*TV*)	5	1.225	0.330	0.088	0.179	0.494
One-breed						
Error	5	3.114	0.671	0.019	0.630	0.702
Genotype × environment	5	1.038	0.128	0.015	0.097	0.155
Genotype	5	1.117	0.120	0.020	0.087	0.160

Prior degrees of freedom (υ) and scale parameter ($S^{2}$), and posterior summary statistics of each variance component obtained from the Gibbs sampler implemented by Stan software for a multibreed or one-breed model.

**Table A4.2:** Data set 2

					HPD95	
Variance component	υ	S^2^	Mean	SD	LOWER	UPPER
Two breeds						
Error	5	0.445	0.418	0.009	0.401	0.437
*Ae. tauschii* (*D*)	5	0.226	0.118	0.025	0.071	0.166
*T. turgidum* (*T*)	5	0.223	0.100	0.020	0.020	0.139
One-breed						
Error	5	0.445	0.417	0.010	0.399	0.434
Genotype	5	0.453	0.233	0.019	0.199	0.274

Prior degree of freedom (υ*)*, parameter ($S^{2}$) and posterior statistics of each variance component of the model. Mean and standard deviation (SD). HPD95 = 95% high posterior density intervals from the Gibbs sampler implemented in Stan software for two breeds and one-breed.
